# Transferability of the Mediterranean Diet to Non-Mediterranean Countries. What Is and What Is Not the Mediterranean Diet

**DOI:** 10.3390/nu9111226

**Published:** 2017-11-08

**Authors:** Miguel Ángel Martínez-González, Maria Soledad Hershey, Itziar Zazpe, Antonia Trichopoulou

**Affiliations:** 1 Department of Preventive Medicine and Public Health, University of Navarra, 31008 Pamplona, Spain; mamartinez@unav.es (M.Á.M.-G.); mhershey@alumni.unav.es (M.S.H.); 2 CIBER Fisiopatología de la Obesidad y la Nutrición (CIBEROBN), Instituto de Salud Carlos III, 28029 Madrid, Spain; 3 Department of Nutrition, Harvard School of Public Health, Boston, MA 02115, USA; 4 IdiSNA, Navarra Institute for Health Research, 31008 Pamplona, Navarra, Spain; 5 Department of Nutrition, Food Science and Physiology, University of Navarra, 31008 Pamplona, Spain; 6 Hellenic Health Foundation, 11527 Athens, Greece; atrichopoulou@hhf-greece.gr; 7 WHO Collaborating Center for Nutrition and Health, Unit of Nutritional Epidemiology and Nutrition in Public Health, Department of Hygiene, Epidemiology and Medical Statistics, University of Athens Medical School, 15772 Athens, Greece

**Keywords:** Mediterranean diet, cardiovascular disease, dietary patterns, dietary intervention, dietary recommendations

## Abstract

Substantial evidence has verified the Mediterranean diet’s (MedDiet) nutritional adequacy, long-term sustainability, and effectiveness for preventing hard clinical events from cardiovascular disease (CVD), as well as increasing longevity. This article includes a cumulative meta-analysis of prospective studies supporting a strong inverse association between closer adherence to the MedDiet and the incidence of hard clinical events of CVD. The MedDiet has become an increasingly popular topic of interest when focusing on overall food patterns rather than single nutrient intake, not only in Mediterranean countries, but also globally. However, several myths and misconceptions associated with the traditional Mediterranean diet should be clearly addressed and dispelled, particularly those that label as “Mediterranean” an eating pattern that is not in line with the traditional Mediterranean diet. The transferability of the traditional MedDiet to the non-Mediterranean populations is possible, but it requires a multitude of changes in dietary habits. New approaches for promoting healthy dietary behavior consistent with the MedDiet will offer healthful, sustainable, and practical strategies at all levels of public health. The following article presents practical resources and knowledge necessary for accomplishing these changes.

## 1. Introduction

The Mediterranean diet (MedDiet) is a scientific concept that reflects the traditional dietary pattern that prevailed in the olive tree-growing areas of the Mediterranean basin before the mid-1960s, that is, before globalization had its influence on lifestyle, including diet [1]. In the current context of assessing the health effects of overall food patterns instead of single nutrients or foods, the MedDiet has become a scientific topic of high interest due to evidence that has directly supported substantial health benefits, including some large trials with hard clinical endpoints [2,3,4,5,6,7,8,9], but this high-quality evidence is not available for any other dietary pattern.

The MedDiet is characterized by its relatively high total fat intake (from olive oil) that makes it palatable, but low in saturated fat and rich in nutrients and dietary fiber content. It is a diet that is rich in antioxidant compounds and bioactive elements with anti-inflammatory effects, and it has a low glycemic index. These health properties help to meet nutritional requirements [10], reach and maintain a healthy body weight [11,12,13], increase longevity [5,9,14], and reduce the risk for chronic disease, including cardiovascular disease (CVD) [1,2,3,4,5,6,7,8,9], type 2 diabetes [15], obesity [11,12,13], metabolic syndrome [16,17], certain cancers [18,19], and cognitive impairment [20,21]. Several important meta-analyses have systematically assessed and reported important health benefits derived from a closer conformity with the MedDiet [5,6,8,9,13,15,16,17,18], but none of them have adopted a cumulative approach to assess the temporal sequence in the accrual of this evidence.

Poor diet quality is a primary cause for chronic disease and mortality in the United States (US) and the adoption of the MedDiet might be instrumental for reversing this situation. The 2015–2020 Dietary Guidelines for Americans stated that about three-fourths of the population followed an unhealthy eating pattern and half of the population was at the limit or exceeding total refined grains and unhealthy sources of protein [22,23,24,25]. The average American diet is also exceeding the recommendations for added sugars, saturated fats, sodium, and calories [22,23,24,25], and is low in micronutrients; particularly potassium, calcium, vitamin D, and fiber. As reported in 2015 by the National Health and Nutrition Examination Survey (NHANES), fast food consumption, far away from the standards of a healthy diet, made up 10–20% of the average American daily calorie intake, [25]. In this context, several observational studies and a few clinical trials have examined the association between this food pattern and different health outcomes. The most important end-point is CVD, because it is the main cause of death. There is a need to update the cumulative evidence on the prevention of CVD by MedDiet, within the framework of a quantitative meta-analysis. To our knowledge, no previous cumulative meta-analysis for this association has been conducted.

In this report, we present a cumulative meta-analysis to quantify the association between adherence to the MedDiet and the risk of mortality from or incidence of CVD and to assess the consistency of previous observational findings with those of randomized controlled trials (RCT). Besides, we argue that the health properties, palatability, potential for sustainability, and nutritional adequacy [1,10] of the MedDiet could be an effective, feasible, and sustainable solution for improving the dietary habits of the American population, and we provide a practical means to transfer this dietary pattern to Non-Mediterranean settings, especially the US, and consequently to address these pressing public health concerns.

## 2. Material and Methods

We conducted electronic searches in PubMed, Embase, Google Scholar, and Web of Science with these search terms: “Mediterranean diet” in combination with keywords relating to cardiovascular events (“CVD”, or “cerebrovascular”, or “cardiovascular” or “ischemic”, or “stroke”, or “coronary”). We also reviewed the bibliographies of the extracted original articles and reviews to locate additional publications. The parameters of search strategy included the following filters: language (English, Spanish, Italian, French), age (18 years or older), and human studies. No time period limit was established. Originals published up until May 2017 were included in this search.

The inclusion criteria were: clinical trials or prospective cohort studies with appropriate control for confounding, original articles, and primary prevention of mortality or incidence of CVD through the MedDiet; the exposure of interest was the adherence to the MedDiet; and, the outcome was mortality from CVD or incidence of cardiovascular events (coronary heart disease or stroke).

On the other hand the exclusion criteria were: presence of previous CVD; reviews, editorials, comments, letters without sufficient data; abstracts of meeting presentations; non-human studies; cross-sectional or case-control studies; studies that did not specifically considered the adherence to the MedDiet on cardiovascular incidence or mortality from CVD, or for which estimates for MedDiet associations were not available; and, studies of other exposures or studies reporting outcomes of other diseases and studies that have studied the adherence to the MedDiet using factor analysis.

Two independent reviewers initially conducted the search strategy on primary titles and abstracts to select potential articles. Next, one of the reviewers assessed in detail the selected full-text articles and decided their eligibility according to the inclusion/exclusion criteria and extracted the data of interest for the cumulative meta-analysis after discussion and consensus.

The following study characteristics were collected: authors, study design, sample size and sample characteristics, dietary assessment method, average duration of follow-up, number of non-fatal and fatal events, and results and covariates in the fully adjusted model.

Finally, the analyses plan was to pool the relative risks and 95% confidence intervals as estimates of the magnitude of association for all of the studies, and the odds ratios or hazard ratios were considered as equivalent to relative risks. Because different cut-off points and scores for adherence to the MedDiet and different categories were present in different articles, we computed a relative risk with 95% confidence interval for an increase of two points in adherence to the MedDiet Scores proposed by Trichopoulou et al. [2], ranged from 0 (minimum adherence) to 9 (maximum adherence). Finally, papers published by the same research group and studying similar factors in the same cohort were checked for potential duplicate data. When it occurred, the most recent set was included in the meta-analysis, but excluding the incident events or cases of mortality reported in previous research.

Other methodological aspects of this meta-analysis are included in the Supplementary Materials.

## 3. Results

**Figure 1:**
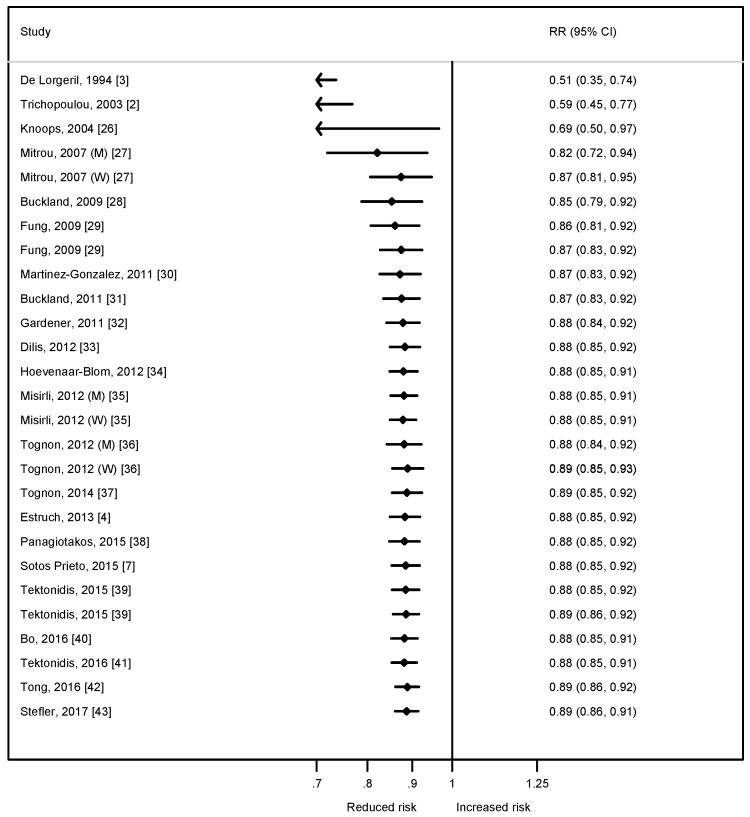
Cumulative meta-analysis of prospective cohort studies of Mediterranean diet adherence (for each 2 additional points in a 0 to 9 score) and the risk of mortality from or incidence of cardiovascular disease. W: Women; M: Men.

Figure 1 shows the cumulative meta-analysis (sorted by the year of publication) of prospective studies (observational cohorts and trials) supporting a strong inverse association between closer adherence to the MedDiet and the incidence of hard clinical events of CVD.

**Figure 2:**
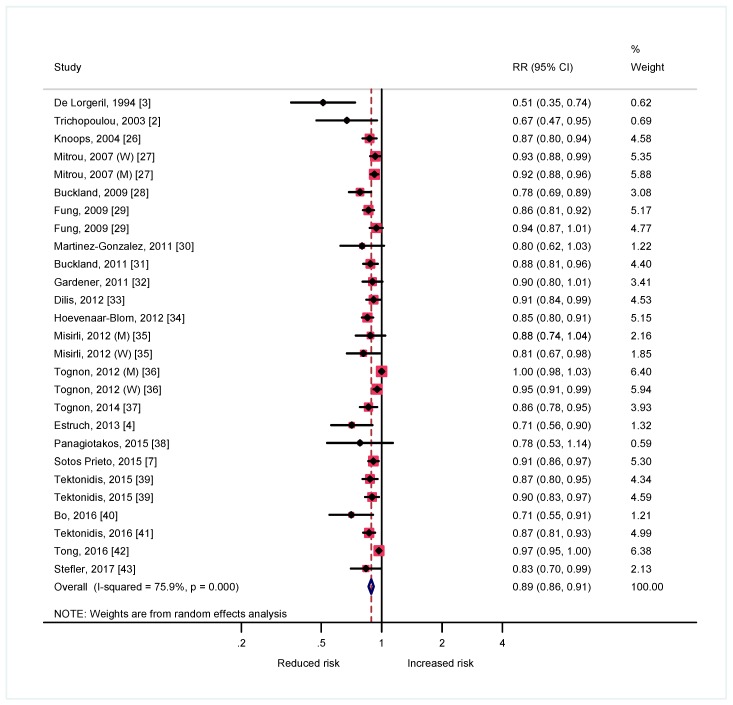
Forest plot describing the association between adherence to MedDiet (for each 2 additional points in a 0 to 9 score) and the risk of mortality from or incidence of cardiovascular disease (CVD). The center of each square indicates the relative risk of the study and the horizontal lines 95% Confidence Intervals (CIs). The area of the square is proportional to the size of the study. The diamond indicates a pooled estimate.

In Figure 2 with 27 estimates showed that each 2-point increment in a 0–9 score of MedDiet was associated with a 11% relative reduction in the risk of CVD (risk ratio: 0.89; 0.86–0.91).

Main prospective observational studies and randomized controlled trials published until May 2017, assessing the effects of the MedDiet on mortality from or incidence of CVD are included in the Supplementary Materials (Table S1). We included the Lyon trial (assessing survivors of a myocardial infarction) because of its importance as a pioneering randomized trial.

Despite the apparent relevance of the two available randomized trials (Lyon Heart Study and PREDIMED) their respective weights only represent 0.62% and 1.32% of the total evidence (Figure 2).

## 4. Discussion

Our cumulative meta-analysis using statistical techniques to synthesize the available evidence from several studies into a single quantitative estimate or summary effect size, which is continually updated each time that a new prospective study was published provides a better picture than other previous attempts to visualize the enormous accrual of evidence supporting the cardiovascular benefits of the MedDiet. In addition, it is challenging to address many different issues that are related to the definition and composition of the Mediterranean diet, particularly with respect to its transferability to the US and other Non-Mediterranean settings. We discuss these issues in the following paragraphs.

### 4.1. What Is the Mediterranean Diet?

Several scoring systems have been used to operationally describe the traditional MedDiet [1,2,8,14,44,45]. Table 1 shows two frequently used scores selected because: (1) the MedDiet Score was the first operational score that appeared in the literature, and is one of the most frequently used scores, with generally consistent results regarding MedDiet-disease associations evaluated so far [2,5,6,44], and (2) the 14-item PREDIMED screener was used in the largest trial of MedDiet for preventing CVD, the PREDIMED (PREvencion con DIeta MEDiterranea) trial [4,45].

The MedDiet Score [2,5,14,44] is based on sample characteristics, with some modifications applied in Europe and the US. Assessments using this score have shown beneficial associations with health outcomes in large epidemiological studies [2,5,6,8,9,14,15,18,44]. One such modification incorporates polyunsaturated fat, in addition to the monounsaturated fat content, to the beneficial high unsaturated:saturated fat ratio, instead of using the monounsaturated:saturated fat ratio that was originally proposed by Ancel Keys [1]. But this definition represents some deviation from the original concept of the traditional MedDiet [7]. This score has been used in a variety of countries and it also may be included within the items “fruit and nuts” and “vegetables” different varieties of local fruit and vegetables that were not available in the olive growing areas of the Mediterranean basin in the 1960’s. A potential inconvenience of this score is that the medians (or other quantiles) that are used as cut-offs are dependent on the sample characteristics and can compromise between-study comparisons or its generalizability. Nonetheless, using medians instead of a priori defined cut-off points is more consistent with the fact that most dietary assessment have been done using food frequency questionnaire, which are tools better suited to rank individuals rather than to accurately measure absolute intakes.

The PREDIMED brief 14-item screener [45] uses pre-defined goals for the consumption of specific food items and overcomes the potential lack of external comparability that is related to the use of sample-specific medians. Additionally, this questionnaire allows for a quick assessment, intervention, and immediate provision of feedback in intervention studies. An interesting literature-based adherence score was proposed by Sofi et al. in 2014. They took into account the means of the items included in the definition of MedDiet in each cohort study and calculated a weighted median of theses means using the sample sizes as weights. In this way they standardized the cut-off points of each food group. However, disparities in categorization of foods are still a limiting factor for the generalizability of results [5].

Regardless of the operational score used, the traditional MedDiet is characterized by a high intake of vegetables, fruits, nuts, legumes, and mainly unrefined, minimally processed cereals; an abundant fat intake from virgin olive oil, through salads, traditionally cooked vegetables, and legumes; a moderate consumption of fish and shellfish, a low consumption of meat and meat products; and, the consumption of wine during meals. Fermented dairy products (cheese and yogurt) are allowed in moderate amounts.

The main sources of fat and alcohol are primarily virgin olive oil (VOO) and wine, respectively. They contain hydroxytyrosol and tyrosol, oleocanthal, resveratrol, and many other dietary bioactive phenolic compounds with substantial anti-inflammatory properties [46]. The anti-atherogenic properties of olive oil were supposedly attributed to its high oleic acid content [47]. However, in recent years, converging evidence indicates that bioactive polyphenols are only present in the extra virgin olive oil (EVOO) and may substantially contribute to these benefits [48]. The concentration of phytochemicals in oils is influenced by the oil extraction procedures. EVOO is obtained from the first pressing of the ripe fruit and has a high content of antioxidants (tocopherols, polyphenols, and phytosterols). Lower-quality oils (refined olive oils) lose antioxidant capacity because they are obtained by physical and chemical procedures, despite their similarity to the extra-virgin variety’s fatty acid composition. The phenolic compounds in EVOO have shown strong antioxidant and anti-inflammatory activity in vitro and in vivo [46,49], and some of their specific metabolites, when objectively measured, have been associated with reduced risk of hard cardiovascular events [50].

The abundant consumption of fruits and vegetables with low caloric value but rich in nutrients allows for a greater intake and ensures long-term adherence to this healthy dietary pattern and nutritional adequacy without vitamin supplements. The use of virgin olive oil, as well as many herbs, increases the palatability of plant-based dishes and facilitates a very high consumption of fresh and tasty vegetables. The seasonality, biodiversity, nutrient density, and the use of a variety of traditional and local food products, as well as culinary traditions, are important elements of the MedDiet. In addition to being a sustainable diet on an individual level, by allowing flexibility in food selection and assuring accessibility to staple ingredients without relying on special diet foods, it is also sustainable for the planet [51]. With a low environmental impact, this dietary pattern contributes to food security and a healthy life for present and future generations. This is due to the MedDiet being a mainly plant-based diet, which implicates smaller water and energy footprint as well as less land usage and fewer greenhouse gas emissions when compared to other dietary patterns. Furthermore, legumes absorb nitrogen and are present in the nitrogen fixation cycle contributing to a natural fertilization system requiring less fertilizer use [51,52,53].

### 4.2. What Is Not the Mediterranean Diet?

In a globalized world, the current dietary pattern in Mediterranean countries has deviated from former traditional foods and culinary traditions [54]. Not all foods consumed nowadays in Mediterranean countries are good examples of a heart-healthy diet. Some prevalent myths and misconceptions on the MedDiet require clarification, since the main challenges for its transferability to the US derive from these misconceptions. They are based on popular definitions of the MedDiet that promote some foods but tend to ignore everything about the foods that are not at all in line with the MedDiet and should be restricted or avoided.

First, the traditional MedDiet is not a purely vegetarian diet; it is primarily, but not exclusively, a plant-based diet that allows for a low consumption of meat and meat products, fermented dairy, and a moderate consumption of fish [1]. Second, the American-style pizza that is widely consumed in the US, despite its Italian name, is not a traditional Mediterranean food; instead, it should be considered as another type of fast food, being one of the top sources of calories, sodium, and saturated fat in the US due to the preparation methods and toppings employed [24]. Third, the main concern regarding alcoholic beverages is not the mere amount of ethanol intake, but rather the pattern of consumption, in opposition to the current habits of the American population. For adults, a traditional MedDiet may include moderate alcohol consumption, always during meals, preferably wine, spread out over the week, with low or no liquor consumption [54]. Of course, binge drinking and preference for beer instead of wine are not part of the traditional MedDiet. Fourth, although there are many reasons to think that the consumption of avocado is healthy, this food is a fruit originating from South America and not a traditional MedDiet food. Fifth, other foods misclassified include some saturated fat-rich dessert preparations, quinoa, margarine, potatoes, tofu, and potato chips. Sixth, a “Japo-Mediterranean” diet, comprised of olive oil, wine, fish, beans, nuts and seeds, soy, vegetables, fruits, bread, rice, seaweed, dairy products, and mushrooms, is not a traditional MedDiet because soy is not a traditional Mediterranean food. Seventh, an “Indo-Mediterranean diet”, which is rich in whole grains, fruits, vegetables, walnuts, mustard, oil, and almonds is not either. Using other unsaturated fat cooking oils other than olive oil, such as flaxseed, peanut, corn, or sunflower oils, with similar or higher content in saturated fat than olive oil, do not pertain to the traditional MedDiet. Although alternative liquid oils (sunflower, canola, soya, or other seeds) that are rich in polyunsaturated lipids are better than lard or butter, they are uncharacteristic of the traditional MedDiet. The proper introduction of the traditional MedDiet in non-Mediterranean countries requires the use of olive oil as the main culinary fat. Some systematic reviews on the MedDiet misleadingly defined the MedDiet as any diet that meets at least two of nine traditional MedDiet characteristics (high consumption of olive oil, legumes, cereals, fruits and vegetables, moderate to high consumption of fish, low consumption of meat and meat products, and moderate consumption of dairy products, mostly as cheese and yogurt, and wine). This definition based on at least two of these characteristics completely lacks specificity, and therefore it may prove useless. Other misleading definitions of the MedDiet are based on macronutrient intake and may indirectly suggest that the MedDiet is defined solely by an unrestricted fat content. This is incorrect, however, a “low fat” MedDiet is not the traditional MedDiet either. The usual amount of fat in the MedDiet is 30–45%, but the important factor is not the amount, but the type of fat: olive oil, nuts, and fatty fish should be the main sources of fat, especially extra-virgin olive oil, which may represent 15% or more of the total caloric intake.

In summary, adoption of the MedDiet inevitably requires a low consumption (both in quantity and frequency), or even null consumption of red meat, processed meats, sweet desserts, and processed foods rich in sugars and fats.

### 4.3. How the American Population Can Adopt the Mediterranean Diet to Their Culture and Lifestyles

The transferability of the traditional MedDiet to the US and other Non-Mediterranean settings is desirable based on its health benefits, also shown in non-Mediterranean countries [1,55,56]. The 2015–2020 Dietary Guidelines for Americans have already taken steps towards adapting the MedDiet for the American population. They include an alternative healthy Mediterranean-style eating pattern, complemented with recommended amounts for each food group in the American customary system. The Harvard School of Public Health, Oldways, a nonprofit food think tank, and other institutions offer practical resources to apply the traditional MedDiet in American settings: cookbooks, blogs, news articles, restaurants, and several hospitals and food chains are promoting this healthful dietary pattern and inspiring Mediterranean culinary practices in the American kitchen [55,56].

The transferability of the MedDiet may seem challenging, but there are means to overcome this challenge (see below) by teaching specific practical recommendations to shift the American food pattern to a cardio-protective MedDiet, as shown in Table 2. Replacing bagged processed snacks with healthier options (mixed tree nuts, fruits, and vegetables); replacing soda and juices with water and moderate amounts of red wine for adults; and, regularly consuming fresh fruit as the usual dessert and setting aside sweets, ice-creams, baked goods, and high fat dairy products, saving them only for occasional celebrations. Significantly reducing snacking in-between meals throughout the day is a cultural lifestyle factor that requires special attention. Substituting red or processed meats with seafood, legumes, and nuts could considerably improve diet quality. Eggs are not included in the main definitions of the traditional MedDiet, and the Dietary Guidelines for Americans 2015–2020 do not include restrictions on egg consumption and do not consider dietary cholesterol as a nutrient of concern. Conversely, other noteworthy recommendations are limiting processed foods, which are typically high in sodium and sugar, as well as breaded and deep fried foods, which are frequently consumed in the US today and are contradictory to the traditional MedDiet. The MedDiet pyramid can serve as a guide to Americans of this healthy diet, including lifestyle behaviors. When considering the multiethnic population of the US, the transferability of the MedDiet should not result as difficult as it has already been shared by several cultures, religions, and traditions. Adopting the MedDiet can be done in an economically affordable way by simply making better food choices and never forgetting the aspect of frugality inherent to the MedDiet. Motivating the public interest and teaching skills for cooking in the Mediterranean style are also fundamental needs. This way, not only will this dietary pattern be accepted for its nutritional value, but also for its delicious taste [1].

By applying evidence-based knowledge and new policy strategies based on the traditional MedDiet, the American government can facilitate the lifestyle changes that are needed to improve public health. Some health initiatives implemented in the US are already showing progress nationally and offer potential hope for future development towards a healthier food system. A good example is the improvement in trans-fat regulation, accomplished by modifying food preparation with different cooking oils. Moreover, food preparation should be better adjusted to the Mediterranean culinary style to improve nutrient intake. This is especially needed in restaurants and in packaged foods, because 32% of Americans’ total daily calories are eaten away from home [23,24,25]. Successful efforts in this direction can follow the path of the American Heart Association’s Heart-Check Program, which has driven part of the fast-food industry to meet nutrient quality standards [23,24,25]. Additionally, a mark label for traditional Mediterranean foods and recipes might facilitate a better adherence to this dietary pattern when making food choices, comparable to the Guiding Stars system in Canada, which observed better food-purchasing patterns and measurable nutritional benefits [57]. Information on websites, blogs, books, health-related resources, and cooking magazines can promote the MedDiet for Americans. Advertising is an enormously influential tool and should be more widely used for promoting health consciousness and high-quality food patterns. Public service announcements can introduce the Mediterranean lifestyle on television, radio, and social media pages. The American culture follows celebrities and social leaders, therefore a spokesperson capable of capturing the attention of Americans and gaining popularity could greatly contribute to the transferability of the MedDiet. Due to the use of local products in the MedDiet, the cultivation of local products contributes to a sustainable environment, employment of local people, and the balance between the territory and the people. Future changes working towards influencing food-purchasing behaviors at supermarkets, farmer’s markets, and food selection on Mediterranean-style menus will need to consider the consumer acceptance to be successful [58]. Nonetheless, if the public health system of the US is successful in fostering a healthier lifestyle and a high-quality dietary pattern closer to the MedDiet, the general public will be more likely to accept this diet.

All of these structural interventions should be coupled with the appropriate personalized health education by clinicians to their patients in an opportunistic approach [59]. The practical clues provided in Table 2, if they are widely, wisely, and timely used, can prove very useful to increase adherence to the MedDiet in the American population at large.

### 4.4. Further Research

More research on the transferability and effectiveness of the MedDiet to the US and its health benefits in non-Mediterranean populations is needed. There is a need for a trial similar to PREDIMED tailored to the American culture employing the overall dietary pattern approach rather than isolated nutrients. In this context, it is important to highlight that the effectiveness of the PREDIMED trial is not to be attributed mainly to the supplemented foods (nuts and olive oil) but to adherence to the full food pattern [60,61]. In order to compare the effectiveness of the transferability of the MedDiet to American settings, randomized intervention studies in which the participants are prescribed and educated on the MedDiet and provided key food items for free would obtain the greatest level of evidence. The National Heart, Lung, and Blood Institute is precisely planning to fund such a trial in the US [55]. This initiative is very much welcomed and it will represent a sound foundation for the transferability of the MedDiet (including extra virgin olive oil and tree nuts) to the US. Studying validated dietary data from previous studies and applying a standard MedDiet adherence scoring system, with only some minor adaptations to be suited to the dietary habits of the target population can also influence dietary guidelines and nutrition policy towards the MedDiet in an evidence-based manner [62]. In addition, beyond the development of a PREDIMED-like trial in the US, further research is also required to find the best way to encourage the public in Non-Mediterranean countries to change their habits towards a more Mediterranean-style diet.

## 5. Conclusions

The traditional MedDiet is a high-quality dietary pattern. The essential elements of the MedDiet include abundant use of extra-virgin olive oil for all culinary purposes, high consumption of plant-derived foods (fresh fruits, vegetables, legumes, tree nuts), moderate-to-high consumption of fish, whole-grain cereals and red wine (with meals). But reduced (or even no consumption at all) of sugar-sweetened beverages, of red and processed meats, milk, butter, whole-fat dairy, sweets, biscuits or cakes. The usual dessert is fresh fruit instead of ice-creams, pies, sweets or creamy desserts. Potatoes or eggs do not enter into the definition of the MedDiet. Other supposedly healthy foods, such as soya, quinoa, flaxseed oil, or other liquid oils different from olive oil are not part of the MedDiet.

No other food pattern is backed by such an important and consistent accrual of sound prospective epidemiological and trial-based evidence supporting the reduction in clinical cardiovascular events.

The transferability of the MedDiet to non_Mediterranean countries can incorporate flexibility but it needs to incorporate all its traditional components. Importantly, a substantial reduction (or even total avoidance) of elements which are fully in opposition to the concept of the traditional MedDiet should be especially stressed to obtain this transferability.

## Figures and Tables

**Table 1:** Two frequently used operational definitions of the Mediterranean diet.

	Mediterranean Diet Score (0 to 9 Points)	PREDIMED Screener Score (0 to 14 Points)
**Positively weighted items**	Monounsaturated/Saturated fat ratio * Vegetables * Fruits and nuts * Legumes * Fish * Cereals *	Olive oil as main culinary fat ≥4 tablespoon/day olive oil ≥2 servings/day vegetables ≥3 servings/day fruits ≥3 servings/week legumes ≥3 servings/week fish
**Negatively weighted items**	Meat/meat products † Dairy products †	≥3 servings/week nuts ≥2 servings/day olive oil sauce with tomato garlic and onion (“sofrito”) Preference for poultry > red meats ‡ <1/day Red/processed meats <1/day Butter/Margarine/cream <1/day carbonated/sugared sodas <2/week commercial bakery, cakes, biscuits or pastries
**Moderate alcohol intake**	5–25 g/day (women) 10–50 g/day (men)	≥7 glasses/week of wine

* One point if the consumption was at or above the sex-specific median. † One point if the consumption was below the sex-specific median. ‡ The wording of the question was: Do you prefer to eat chicken or turkey instead of beef, pork, hamburgers of sausages?

**Table 2:** Practical approaches for adopting the Mediterranean Diet.

Mediterranean Diet	Western Diet	Incorporating the Mediterranean Diet
Olive oil	Solid fats; butter, margarine, cream cheese, coconut, palm, and tropical oils Cooking oils; soybean, canola, corn, sunflower	Use extra virgin or virgin olive oil, if not always possible, prefer using olive oil raw Consume with vegetables and legumes in many salads, stir fries and sautés Use herbs, spices, garlic, onions and lemon for flavor when cooking
Vegetables	Starchy vegetables predominate over lower calorie vegetables Low/under consumption	Always try incorporating vegetables at lunch and dinner, often as main dish Aim for ≥2 servings/day 1 servings day should be consumed raw, adequately dressed with extra-virgin olive oil and vinegar, preferably in salads
Fruits	Low/under consumption Fruit products with added sugars	Serve fresh raw fruits as the usual dessert with the exception of feasts and celebrations Aim for ≥3 servings/day of fresh fruits Variety and temporality
Whole grains; Bread	White refined flour Refined and processed cereals Sugary breakfast cereals Pizza rich in flour and cheese Sliced bread; includes butter and sugar; higher caloric form of bread	Switch to whole grain bread, pasta, rice, and flour Try making homemade pizza with olive oil, less cheese and topped with fresh vegetables to create a Mediterranean-style pizza Try drizzling toast with extra virgin olive oil for breakfast or a snack
Legumes	Low/under consumption High sodium in canned products	Consume ≥3 servings week any variety of legumes such as any variety of beans, lentils, chickpeas, peas
Seafood; Fish	Low/under consumption Lack of variety Expensive	Aim for ≥1 servings/week white fish (cod, flounder, tilapia), ≥2 servings/week fatty fish (tuna, salmon, sardines) and occasional shellfish (oysters, clams, squid, shrimp) Wild-caught, farm-raised, fresh, frozen, or canned fish or seafood are all acceptable options
Meat; Poultry	Red meat consumed regularly; beef, pork, processed meats (cold cuts, sausages, hot dogs, hamburgers, etc.) Large portionsDaily consumption	Preferably choose lean poultry; chicken and turkey Moderate portion sizes (3–4 oz.) Save red meat for occasional consumption; 1–3 servings/month
Dairy: yogurt and cheese	Various and abundant amounts of dairy products; milk, processed cheese, cream cheese, ice cream, milkshakes	Regular or fat-free natural yogurt (add nuts and fruit for flavor), but never use yogurt to replace fresh fruit as dessert Avoid the excessive consumption of ice-cream prevailing in the US. Occasional consumption of cheese; both fresh and cured cheeses in *small* portions
Nuts and olives	Butter, margarine, ketchup, mayonnaise dips, cream sauces, dressings Processed prepackaged snacks	Primary source of fat should be extra-virgin olive oil and olives Consume a handful of raw nuts a day, or ≥3 servings/week, as a healthy replacement for processed snacks Consume olives as a snack or in salads Walnuts, almonds, hazelnuts, pistachios, etc.
Homemade baked goods	Industrial store-bought baked goods (cakes, cookies, pies, brownies, donuts) Creamy and sugary desserts (candy, pudding, syrups)	Rather than buying baked goods, occasionally bake at home using olive oil instead of butter Consume baked goods and high fat dairy products occasionally
Wine	Beer, liquor, sugar sweetened drinks (soft drinks, sports drinks, juices, flavored water) Heavy/binge drinking	Replace beer or liquors with wine, preferably red wine, no more than 2 glasses (10 oz.)/day for men and 1 glass (5 oz.)/day for women consume always with a meal Replace soda and juices with water

## Supplementary Materials

The following are available online at www.mdpi.com/2072-6643/9/11/1226/s1, Table S1: Main prospective observational studies and randomized controlled trials published until May 2017, assessing the effects of the Mediterranean diet on mortality from or incidence of cardiovascular disease.
