# Correction: Martínez-González, M.A. et al. Transferability of the Mediterranean Diet to Non-Mediterranean Countries. What Is and What Is Not the Mediterranean Diet. *Nutrients* 2017, *9*, 1226

**DOI:** 10.3390/nu10070823

**Published:** 2018-06-26

**Authors:** Miguel Ángel Martínez-González, Maria Soledad Hershey, Itziar Zazpe, Antonia Trichopoulou

**Affiliations:** 1 Department of Preventive Medicine and Public Health, University of Navarra, 31008 Pamplona, Spain; mamartinez@unav.es (M.Á.M.-G.); mhershey@alumni.unav.es (M.S.H.); 2 CIBER Fisiopatología de la Obesidad y la Nutrición (CIBEROBN), Instituto de Salud Carlos III, 28029 Madrid, Spain; 3 Department of Nutrition, Harvard School of Public Health, Boston, MA 02115, USA; 4 IdiSNA, Navarra Institute for Health Research, 31008 Pamplona, Navarra, Spain; 5 Department of Nutrition, Food Science and Physiology, University of Navarra, 31008 Pamplona, Spain; 6 Hellenic Health Foundation, 11527 Athens, Greece; atrichopoulou@hhf-greece.gr; 7 WHO Collaborating Center for Nutrition and Health, Unit of Nutritional Epidemiology and Nutrition in Public Health, Department of Hygiene, Epidemiology and Medical Statistics, University of Athens Medical School, 15772 Athens, Greece

The authors have requested that the following changes be made to their paper [1].

In Table 1, page 5, two frequently used operational definitions of the Mediterranean diet are presented. There is a typographical error in one of the items of the PREDIMED screener score for the consumption of “sofrito”. Instead of saying two or more times per week, it said two or more times per day. More can be read on this score in the original article that defines this short screener [2]. In Table 1, “tablespoon” was replaced with “tablespoons”. Meanwhile, in the footer of Table 1, “hamburgers of sausages” was replaced with “hamburgers, or sausages”. The table should read as the following.

The authors apologize to the readers for any inconvenience caused by the change. Although this typographical error may have misled the reader on the operational definition of the Mediterranean diet according to the PREDIMED screener score, it does not affect the scientific results. The original manuscript will remain online on the article webpage, with a reference to this Correction.

## Figures and Tables

**Table 1:** Two frequently used operational definitions of the Mediterranean diet.

	Mediterranean Diet Score (0 to 9 Points)	PREDIMED Screener Score (0 to 14 Points)
**Positively weighted items**	Monounsaturated/Saturated fat ratio * Vegetables * Fruits and nuts * Legumes * Fish * Cereals *	Olive oil as main culinary fat ≥4 tablespoons/day olive oil ≥2 servings/day vegetables ≥3 servings/day fruits ≥3 servings/week legumes ≥3 servings/week fish
**Negatively weighted items**	Meat/meat products ^†^ Dairy products ^†^	≥3 servings/week nuts ≥2 servings/week olive oil sauce with tomato, garlic, and onion (“sofrito”) Preference for poultry > red meats ^‡^ <1/day red/processed meats <1/day butter/margarine/cream <1/day carbonated/sugared sodas <2/week commercial bakery, cakes, biscuits, or pastries
**Moderate alcohol intake**	5–25 g/day (women) 10–50 g/day (men)	≥7 glasses/week of wine

* One point if the consumption was at or above the sex-specific median. ^†^ One point if the consumption was below the sex-specific median. ^‡^ The wording of the question was as follows: “Do you prefer to eat chicken or turkey instead of beef, pork, hamburgers, or sausages?”
