# Supplementary material for: Transferability of the Mediterranean Diet to Non-Mediterranean Countries. What Is and What Is Not the Mediterranean Diet
Source: Nutrients. 2017 Nov 8;9(11):1226. doi: 10.3390/nu9111226 (PMC5707698; doi:10.3390/nu9111226)
Supplement: Supplementary file 1 [file nutrients-09-01226-s001.pdf]

SUPPLEMENTAL MATERIAL

Table 3. Main prospective observational studies and randomized controlled trials published until May 2017, assessing the effects of the Mediterranean diet on mortality from or incidence of cardiovascular disease.

Author, year	Study	Location	Outcome(s)	Exposure	n	Follow-up	Results/observations
De Lorgeril, 1994	Lyon Diet Heart Study	France	CV deaths, non-fatal MI and total mortality	MedDiet rich in alpha-linolenic diet *vs* usual post-infarct prudent diet	605	27 months	In the experimental group the adjusted RR of CV deaths and of total major primary endpoints were 0.24 (95%CI: 0.07-0.85) and 0.27 (95%CI: 0.12-0.59) respectively
Trichopoulou, 2003	Greek population	Greece	Total mortality, CV mortality and cancer mortality	MedDiet (range of score 0-9)	22043	44 months	A higher adherence to MedDiet was associated with lower risk of mortality due CHD [HR=0.67 (95%CI: 0.47-0.94)]
Knoops, 2004	The HALE Project	13 European countries	Mortality from all causes, CHD, CVD and cancer.	MedDiet score (0-8 range) and lifestyle factors	2339	10 years	Adhering to MedDiet was associated with a lower risk of mortality from CVD [HR= 0.71 (95 % CI: 0.58-0.88)] and from CHD [HR= 0.61 (95 % CI (0.43-0.88)
Mitrou, 2007	NIH-AARP Diet and Health Study	US	All-cause, CVD and cancer mortality	MedDiet (9 point score)	214284 men and 166012 women	5 years	The higher adherence to MedDiet was associated with reduced CVD mortality in men [HR=0.78 (95 %CI:0.69-0.87)] and in women [HR=0.81 (95 % CI: 0.68-0.97)]
Fung, 2009	Nurses' Health Study	US	CHD, stroke and CVD deaths	Alternate MedDiet Score (0-9 range)	76522	20 years	Women in the top Alternate MedDiet score were at lower risk for CHD, stroke and CVD mortality [RR=0.71 (95 % CI 0.62-0.82)], [RR=0.87 (0.73-1.02)] and [RR=0.61 (0.49-0.76)] respectively.

Author, year	Study	Location	Outcome(s)	Exposure	n	Follow-up	Results/observations
Buckland, 2009	Spanish EPIC cohort Study	Spain	CHD events	Relative MedDiet score (18-unit)	41078	10.4 years	A 1-unit increase in rMedDiet score was associated with a 6 % reduced risk of CHD (95 % CI: 0.91, 0.97)
Martinez-González, 2011	SUN cohort study	Spain	Composite (CVD death, CHD, MI, revascularization procedures, or fatal or nonfatal stroke)	MedDiet (A 9-point score)	13609	12.3	The highest adherence to the MedDiet exhibited a lower CV risk [HR=0.41 (95% CI: 0.18-0.95)]. For each 2-point increment in the score, the adjusted HR were 0.80 (95% CI: 0.62-1.02)] for total CVD and 0.74 (0.55-0.99)] for CHD
Gardener, 2011	NOMAS cohort	US	Stroke, MI and Vascular death	MedDiet (0-9 scale)	2568	9 years	A point increase of the MedDiet score was inversely associated with risk of vascular death [HR= 0.91 (95% CI 0.85-0.98)]
Buckland, 2011	Spanish EPIC cohort Study	Spain	All-cause and cause-specific mortality	Relative MedDiet score (18-unit)	40622	13.4 years	A 2-unit increase in rMed score was associated with a 12 % decreased risk of CVD deaths [HR=0.88 (95% CI 0.81-0.95)]
Tognon, 2012	VIP study	Northern Sweeden	Total or cause-specific mortality	Modified MedDiet score (0-8 range)	77151		CVD mortality was inversely associated with the modified MedDiet score in women [HR=0.90 (95% CI:0.82-0.99)]
Misirli, 2012	EPIC- Greece	Greece	Incidence of and mortality from cerebrovascular diseases	MedDiet (10-point scale)	23601	10.6	Increased adherence to the MedDiet, as measured by 2-point increments, was inversely associated with cerebrovascular diseases incidence [HR=0.85 (95% CI:0.74-0.96)]. In men [HR=0.88 (95% CI:0.74-1.04)] and in women [HR=0.81 (95% CI:0.67-0.98)]

Author, year	Study	Location	Outcome(s)	Exposure	n	Follow-up	Results/observations
Dilis, 2012	EPIC- Greece	Greece	CHD event	MedDiet (range 0-9 points)	23929	10.6	A 2-point increase in the MedDiet score was associated with lower CHD mortality [HR=0.75 (95% CI: 0.57-0.98)] among women and [HR=0.81 (95% CI: 0.67-0.99)] among men.
Hoevenaar-Blom, 2012	EPIC-NL-Germany	Germany	Incidence of total and specific CVD	MedDiet (range 0-9)	40011	11.8	A 2-unit increment in MedDiet score was inversely associated with fatal CVD [HR=0.78 (95% CI: 0.69-0.88)], total CVD [HR=0.95 (95% CI: 0.91-0.98)], MI [HR=0.86 (95% CI: 0.79-0.93)], stroke [HR=0.88 (95% CI: 0.78-1.00)] and composite CVD [HR=0.85 (95% CI: 0.80-0.91)]
Tognon, 2014	Danish cohort study	Denmark	Total mortality, CV incidence and mortality	Three differente MedDiet scores	1849	13 years	The modified MedDiet score was inversely associated with total mortality and with CVD and MI incidence and mortality.
Estruch, 2013	PREDIMED TRIAL	Spain	CV events and mortality	MedDiet+extra virgin olive oil; MedDiet + nuts; Low fat diet	7447	4.8 years	A MedDiet supplemented with extra-virgin olive oil or nuts reduced the incidence of major CV events [HR=0.70 (95% CI: 0.54-0.92)] and [HR=0.72 (95% CI: 0.54-0.96)] respectively
Sotos-Prieto, 2015	HPFUS NHS	US	CHD and stroke	Several food patterns. Alternative MedDiet score (range 0-9)	80538	17.3 years	Improving adherence to MedDiet during the first 4 years of follow-up associated with a lower risk of CVD [HR=0.91 (95% CI: 0.86-0.97)]

Author, year	Study	Location	Outcome(s)	Exposure	n	Follow-up	Results/observations
Panagiotakos, 2015	ATTICA	Greece	CVD and all-cause mortality	MedDiet (score 0-55) and lifestyles	2583	10 years	MedDiet associated with lower risk of CVD [RR=0.98 (95% CI: 0.95-1.01)], for each additional unit in a 0-55 score, equivalent to a [HR=0.78 (95% CI: 0.53-1.13)] for 2 units in the score
Tektonidis, 2015	SMC	Sweden	MI, stroke, HF	MedDiet (0-8 scale)	32921	10 years	MedDiet associated with lower risk of MI [RR=0.74 (95% CI:0.61-0.90)], HF [RR=0.79 (95% CI:0.68-0.93)] and ischemic stroke [RR=0.78 (95% CI:0.65-0.93)], but not hemorrhagic stroke [RR=0.88 (95% CI:0.61-1.29)].
Tektonidis, 2016	COSM	Sweden	Heart failure	MedDiet (0-8 score)	37308	10.9 years	Inverse association of the MedDiet with heart failure [HR=0.85 (95% CI:0.78-0.91)] for each 2 additional points in the score
Bo, 2016	Turin study	Italy	Mortality and CVD	MedDiet (0-9 scale)	1658	12 years	High adherence to MedDiet inversely associated with all-cause mortality [HR=0.83 (95% CI: 0.72–0.96)] and CVD [HR=0.79, (95% CI:0.65–0.97)]. No association with mortality among high risk subjects
Tong, 2016	UK-based EPIC-Norfolk cohort	UK	Incident CVD, CVD mortality and all-cause mortality	MedDiet score (0-18 range)	23902	17 years	The MedDiet score was significantly associated with lower incidence of the incidence of CVD [HR=0.97 (95% CI:0.94–0.99)]
Stefler, 2017	HAPiEE	Czech Republic, Poland, Russia	Mortality, CVD	MedDiet (0-17 points)	19333	7 years	One standard deviation (SD) in MedDiet adherence inversely associated with all-cause death (HR, 95 % CI 0.93, 0.88–0.98) and CVD (0.90, CI 0.81–0.99)

CV: Cardiovascular. CHD: Coronary Heart Disease. CVD: Cardiovascular Disease. MI: myocardial infarction. MedDiet: Mediterranean Diet.

CI: Confidence Interval. HR: Hazard Ratio. RR: Relative Risk.

Protocol for the cumulative meta-analysis of studies of Mediterranean Diet adherence and the risk of mortality from or incidence of cardiovascular disease

Martínez-González MA[a,b,c,d], Hershey MS[a], Zazpe I[a,b,d,e], Trichopoulou A[e,f]

[a]University of Navarra, Department of Preventive Medicine and Public Health, IdiSNA, Pamplona, Spain.

[b]CIBER Fisiopatología de la Obesidad y la Nutrición (CIBEROBN), Instituto de Salud Carlos III, Madrid, Spain.

[c]Department of Nutrition, Harvard School of Public Health, Boston, USA.

[d]Instituto de Investigación Sanitaria de Navarra (IdiSNA)

[e]University of Navarra, Department of Nutrition, Food Science and Physiology, Pamplona, Spain

[e]Hellenic Health Foundation. Athens, Greece.

[f]WHO Collaborating Center for Nutrition and Health, Athens Medical School

Corresponding author: mamartinez@unav.es; Tef.: +34-636-355-333

INTRODUCTION

Cardiovascular diseases, together diabetes, cancer and other chronic diseases, are among the leading causes of morbidity and mortality in developed and non-developed countries. The traditional Mediterranean diet (MedDiet) pattern has been widely recommended for the prevention of the chronic disease.

Several observational studies and a few clinical trials have examined the association between this food pattern and different health outcomes. However, the evidence on the prevention of cardiovascular disease by MedDiet deserves to be systematically reviewed.

The purpose of this cumulative meta-analysis is to quantify the association between the adherence to the MedDiet and risk of mortality from or incidence of cardiovascular disease and to assess the consistency of previous observational findings with those of randomised controlled trials (RCT).

SEARCH STRATEGY AND STUDY SELECTION

We will conduct electronic searches in PubMed, Embase, Google Scholar and Web of Science.

Search terms: “Mediterranean diet” in combination with keywords relating to cardiovascular events (“cardiovascular disease”, or “cerebrovascular”, or “ischemic”, or “stroke”, or “coronary”). We also will review the bibliographies of the extracted articles and reviews to locate additional publications.

The parameters of search strategy will include the following filters: language (English, Spanish, Italian, French), age (up to 18 years old) and human studies. No time period limit will be established. Originals published up until May 2017 will be included in this search.

INCLUSION AND EXCLUSION CRITERIA

Inclusion criteria

- Clinical trials or prospective cohort studies with appropriate control of confounding, originals and primary prevention of mortality or incidence of cardiovascular disease through MedDiet.
- The exposure of interest was the adherence to the MedDiet
- The outcome was mortality from cardiovascular disease or incidence of cardiovascular events (coronary heart disease or stroke)

Exclusion criteria

- Presence of previous cardiovascular disease
- Reviews, editorials, comments, letters without sufficient data
- Abstracts of meeting presentations
- Non-human studies
- Cross-sectional or case-control studies
- Studies that did not specifically considered the adherence to the MedDiet on cardiovascular incidence or mortality from cardiovascular disease, or for which estimates for MedDiet associations were not available
- Studies of other exposures or studies reporting outcomes of other diseases
- Studies that have studied the adherence to the MedDiet using factor analysis

DATA EXTRACTION

Two independent reviewers initially will conduct the search strategy on primary titles and abstracts to select potential articles. Next, one of the reviewers will assess in detail the selected full-text article and will decide their eligibility according to the inclusion/exclusion criteria and will extract the data of interest for the cumulative meta-analysis after discussion and consensus.

Study details: authors, study design, sample size and sample characteristics, dietary assessment method, average duration of follow-up, number of non-fatal and fatal events and results and covariates in fully adjusted model.

Analyses plan

- Relative risks and 95% confidence intervals will be considered as the magnitude of association for all studies, and the odds ratios or hazard ratios will be considered equivalent to relative risks.
- Those articles reporting both risk of mortality from or incidence of cardiovascular disease will be treated as two separate reports.
- Because it is possible that different cut-off points for adherence to the MedDiet categories will be present in different articles, we will compute a relative risk with 95% confidence interval for an increase of two points in adherence to the MedDiet score for each report.

- Papers published by the same research group and studying similar factors in the same cohort will be checked for potential duplicate data. When it occurs, the most recent set will be used for meta-analysis, but excluding the incident events or cases of mortality reported in previous research.

Management and Coordination

- MAM-G will develop the initial plan, study design, and will be responsible for data collection, data extraction, and data initial interpretation.
- MAM-G will be responsible for statistical analysis.
- MAM-G and IZ will be responsible for data interpretation, critical revision of intellectual content, and approval of the version to be published.

REFERENCES

1. Trichopoulou A, Martínez-González MA, Tong TY, et al. Definitions and potential health benefits of the Mediterranean diet: views from experts around the world. BMC Med 2014;12:112.
2. Trichopoulou A, Costacou T, Bamia C, Trichopoulos D. Adherence to a Mediterranean diet and survival in a Greek population. N Engl J Med 2003;348:2599-608.
3. Estruch R, Ros E, Salas-Salvado J, et al. Primary prevention of cardiovascular disease with a Mediterranean diet. N Engl J Med 2013;368:1279-90.
4. Sofi F, Macchi C, Abbate R, Gensini GF, Casini A. Mediterranean diet and health status: an updated meta-analysis and a proposal for a literature-based adherence score. Public Health Nutr 2014;17:2769-82.
5. Martinez-Gonzalez MA, Bes-Rastrollo M. Dietary patterns, Mediterranean diet, and cardiovascular disease. Curr Opin Lipidol 2014;25:20-6.
6. Sotos-Prieto M, Bhupathiraju SN, Mattei J, et al. Changes in Diet Quality Scores and Risk of Cardiovascular Disease Among US Men and Women. Circulation 2015;132:2212-9.
7. Grosso G, Marventano S, Yang J, et al. A Comprehensive Meta-analysis on Evidence of Mediterranean Diet and Cardiovascular Disease: Are Individual Components Equal? Crit Rev Food Sci Nutr 2015 Nov 3 [Epub ahead of print].
8. Martinez-Gonzalez MA, Martin-Calvo N. Mediterranean diet and life expectancy: beyond olive oil, fruits, and vegetables. Curr Opin Clin Nutr Metab Care 2016;19:401-407.
9. Román-Viñas B, Ribas Barba L, Ngo J, Martínez-González MA, Wijnhoven TM, Serra-Majem L. Validity of dietary patterns to assess nutrient intake adequacy. Br J Nutr 2009;101 Suppl 2:S12-20.
10. Estruch R, Martínez-González MA, Corella D, et al. Effect of a high-fat Mediterranean diet on bodyweight and waist circumference: a prespecified secondary outcomes analysis of the PREDIMED randomised controlled trial. Lancet Diabetes Endocrinol 2016;4:666-76.

11. Trichopoulou A, Bamia C, Trichopoulos D. Anatomy of health effects of Mediterranean diet: Greek EPIC prospective cohort study. BMJ 2009;338:b2337.
12. Esposito K, Maiorino MI, Bellastella G, et al. A journey into a Mediterranean diet and type 2 diabetes: a systematic review with meta-analyses. BMJ Open 2015;5:e008222.
13. Schwingshackl L, Missbach B, König J, Hoffmann G. Adherence to a Mediterranean diet and risk of diabetes: a systematic review and meta-analysis. Public Health Nutr 2015;18:1292-9.
14. Mancini JG, Filion KB, Atallah R, Eisenberg MJ. Systematic Review of the Mediterranean Diet for Long-Term Weight Loss. Am J Med 2016;129:407-415.
15. Nordmann AJ, Suter-Zimmermann K, Bucher HC, Shai I, Tuttle KR, Estruch R, Briel M. Meta-analysis comparing Mediterranean to low-fat diets for modification of cardiovascular risk factors. Am J Med. 2011;124:841-51.e2.
16. Godos J, Zappalà G, Bernardini S, Giambini I, Bes-Rastrollo M, Martinez-Gonzalez MA. Adherence to the Mediterranean diet is inversely associated with metabolic syndrome occurrence: a meta-analysis of observational studies. Int J Food Sci Nutr 2016:1-11. [Epub ahead of print]
17. Garcia M, Bihuniak JD, Shook J, Kenny A, Kerstetter J, Huedo-Medina TB. The Effect of the Traditional Mediterranean-Style Diet on Metabolic Risk Factors: A Meta-Analysis. Nutrients 2016;8:168.
18. Schwingshackl L, Hoffmann G. Adherence to Mediterranean diet and risk of cancer: a systematic review and meta-analysis of observational studies. Int J Cancer 2014;135:1884-97.
19. Schwingshackl L, Hoffmann G. Adherence to Mediterranean diet and risk of cancer: an updated systematic review and meta-analysis of observational studies. Cancer Med 2015;4:1933-47.
20. Toledo E, Salas-Salvadó J, Donat-Vargas C, et al. Mediterranean Diet and Invasive Breast Cancer Risk Among Women at High Cardiovascular Risk in the PREDIMED Trial: A Randomized Clinical Trial. JAMA Intern Med 2015;175:1752-60.
21. Martínez-Lapiscina EH, Clavero P, Toledo E, et al. Mediterranean diet improves cognition: the PREDIMED-NAVARRA randomised trial. J Neurol Neurosurg Psychiatry 2013;84:1318-25.
22. Cao L, Tan L, Wang HF, et al. Dietary Patterns and Risk of Dementia: a Systematic Review and Meta-Analysis of Cohort Studies. Mol Neurobiol 2016;53:6144-6154.
